# Emergency transvenous temporary pacing during rotational atherectomy

**DOI:** 10.3389/fcvm.2023.1322459

**Published:** 2023-12-14

**Authors:** Konstantin Schwarz, Julia Mascherbauer, Elisabeth Schmidt, Martina Zirkler, Gudrun Lamm, Paul Vock, Chun Shing Kwok, Josip Andelo Borovac, Roya Anahita Mousavi, Uta C. Hoppe, Gregor Leibundgut, Maximilian Will

**Affiliations:** ^1^ Karl Landsteiner University of Health Sciences, Department of Internal Medicine 3, University Hospital St. Pölten, Krems, Austria; ^2^ Department of Cardiology, University North Midlands NHS Trust, Stoke-on-Trent, United Kingdom; ^3^ Division of Interventional Cardiology, Cardiovascular Diseases Department, University Hospital of Split (KBC Split), Split, Croatia; ^4^ University Department of Internal Medicine II, Cardiology and Internal Intensive Care Medicine, Paracelsus Medical University, Salzburg, Austria; ^5^ Klinik für Kardiologie, Universitätsspital Basel, Basel, Switzerland; ^6^ Karl Landsteiner Institute for Cardiometabolics, Karl Landsteiner Society, St. Pölten, Austria

**Keywords:** percutaneous coronary intervention, conduction disorders, transvenous temporary pacing, rotational atherectomy, complications, bradycardia, heart block

## Abstract

**Background:**

Rotational atherectomy (RA) during percutaneous coronary intervention may cause transient bradycardia or a higher-degree heart block. Traditionally, some operators use prophylactic transvenous pacing wire (TPW) to avoid haemodynamic complications associated with bradycardia.

**Objective:**

We sought to establish the frequency of bail-out need for emergency TPW insertion in patients undergoing RA that have received no upfront TPW insertion.

**Methods:**

We performed a single-centre retrospective study of all patients undergoing RA between October 2009 and October 2022. Patient characteristics, procedural variables, and in-hospital complications were registered.

**Results:**

A total of 331 patients who underwent RA procedure were analysed. No patients underwent prophylactic TPW insertion. The mean age was 73.3 ± 9.1 years, 71.6% (*n* = 237) were male, while nearly half of the patients were diabetic [*N* = 158 (47.7%)]. The right coronary artery was the most common target for RA (40.8%), followed by the left anterior descending (34.1%), left circumflex (14.8%), and left main stem artery (10.3%). Altogether 20 (6%) patients required intraprocedural atropine therapy. Emergency TPW insertion was needed in one (0.3%) patient only. Eight (2.4%) patients died, although only one (0.3%) was adjudicated as being possibly related to RA-induced bradycardia. Five patients (1.5%) had ventricular fibrillation arrest, while nine (2.7%) required cardiopulmonary resuscitation. Six (1.8%) procedures were complicated by coronary perforation, two (0.6%) were complicated by tamponade, while 17 (5.1%) patients experienced vascular access complications.

**Conclusions:**

Bail-out transvenous temporary pacing is very rarely required during RA. A standby temporary pacing strategy seems reasonable and may avoid unnecessary TPW complications compared with routine use.

## Introduction

Rotational atherectomy (RA) plays a pivotal role in contemporary calcium modification armamentarium during percutaneous coronary intervention (PCI). This technique was first described by Fourier in a series of 12 patients in 1989 (1). Despite technical improvements over time in equipment and technology, the basic characteristics of the procedure have remained largely unchanged. However, with increasing experience among operators, various recommendations of the practical handling of the device have evolved. Compared with bail-out RA PCI strategy, upfront planned RA decreases periprocedural complications (2).

Further, it was noticed that due to the risk of distal embolisation of calcific microparticles and associated neuro-hormonal response, a transient heart block can occur. This reaction was noticed more frequently following RA of the right coronary artery (RCA) or in case of dominant left circumflex artery (CX) (3). Lesion length, burr-to-artery ratio, and total duration of runs were other independent predictors of RA-induced bradycardia (4).

Historically, a routine prophylactic transvenous temporary pacing wire (TPW) was recommended for prevention of symptomatic bradycardia induced by rotational atherectomy. Mitar et al. depicted a small single-centre retrospective observational study (*n* = 134) in which TPW was used in 50% of patients, and heart block or pacemaker (PM) activation in 42 (31%) patients was described (3).

Over the last decade, clinical practice turned away from routine prophylactic TPW insertion prior to procedures involving RA (5, 6). Currently, there is a large paucity of data supporting or disproving this strategy. Concerns of TPW complications, and the relatively short-lived bradycardias and atrioventricular (AV) blocks (which respond quickly to vagolytic manoeuvres) may favour the conservative strategy.

In this single-centre study, we sought to establish the real-life occurrence of significant bradycardia and AV block necessitating TPW insertion during RA. RA is usually performed in coronary lesions with severe calcifications in frail high-risk patients with complex anatomies, and carries higher risk of death, cardiac tamponade, or emergency bypass surgery (7). Predictors of RA PCI complications are patient related (old age, impaired kidney function, or previous myocardial infarction) and procedure related (emergency procedure, triple-vessel disease and low institutional volume) (7). As a secondary outcome, we elected to document other procedural complications related to high-risk PCI including coronary perforation, tamponade, stalled RA burr, cardiac arrest, emergency coronary artery bypass graft (CABG), death, and vascular access complications.

## Methods

This retrospective observational single-centre study was performed at a tertiary hospital centre, the University Hospital Sankt Pölten, Austria. It was approved by the Karl Landsteiner Scientific Integrity and Ethics committee (Ethics commission number 1057/2023) and was performed in compliance with the Declaration of Helsinki. The reporting of this study is in accordance with the STrengthening the Reporting of OBservational studies in Epidemiology (STROBE) recommendations (8).

All patients undergoing RA in our centre between 1 October 2009 and 31 October 2022 were identified from the hospital PCI database. The primary aim of this study was to define the frequency of bradycardia and AV blocks necessitating bail-out TPW insertion during RA procedure.

### Statistical analysis

All analyses were performed using MS Excel 2016 (Microsoft, Redmond, CA, USA) and GraphPad Prism (Version 5.04) software. Descriptive statistics were performed. Categorial variables were expressed as absolute numbers and percentages. Continuous variables were expressed as mean and standard deviation (SD) or median and interquartile range (IQR), depending on the normality of distribution. If a larger number of emergency TPW procedures were to be identified, than an exploratory analysis examining possible predictors for TPW would be performed.

## Results

In total, 331 patients undergoing RA were identified from our hospital database. Prophylactic TPW insertion prior to planned RA in a stable patient has not been routine practice in our centre. Hence, no patient in our database received temporary wire as a primary means of prevention of symptomatic bradycardia prior to RA.

Altogether 237 (71.6%) patients were male, and the majority of procedures were performed in an elective setting (*N* = 207, 60.7%). Twenty-two (6.6%) patients had permanent pacing system implanted before RA. The RCA (40.8%) and left anterior descending artery (LAD) (34.1%) were the most commonly treated target vessels. Femoral access was used in 161 (48%) patients. Detailed demographics of the patient population are provided in Table 1. Intraprocedural complications are listed in Table 2.

**Table 1:** Demographics and procedural characteristics of patients undergoing rotational atherectomy.

Demographics and procedural characteristics (*n* = 331)	*n* (%) or mean ± SD or median [IQR]
Age	73.3 ± 9.1
Male sex, *n* (%)	237 (71.6)
Height, cm (SD)	170.8 ± 6.5
Weight, kg (SD)	89.2 ± 9.8
BMI, mean (SD)	29.9 (2.0)
History of congestive heart failure, *n* (%)	125 (37.8)
LV function, *n* (%)	
Normal	207 (60.7)
Mildly impaired	56 (16.9)
Moderately impaired	40 (12.1)
Severely impaired	28 (8.5)
Valvular pathology	100 (30.2)
Permanent PM/ICD/CRT	22 (6.6)
Diabetes, *n* (%)	158 (47.7)
CKD	70 (21.1)
Creatinine, median [IQR]	0.97 [0.83–1.25]
Stroke/TIA	69 (20.8)
Hypertension	297 (89.7)
Smoker	159 (48.0)
Current	62 (18.7)
Ex-smoker	97 (29.3)
Dyslipidaemia	280 (84.6)
PAD	66 (19.9)
CABG history	64 (19.3)
Multivessel disease	305 (92.1)
CTO	18 (5.4)
Presentation	
Elective	205 (61.9)
NSTEMI/UA	87 (26.3)
STEMI	39 (11.8)
Access	
Radial	163 (49.2)
Femoral	161 (48.6)
Brachial	7 (2.1)
Artery treated with RA	
RCA	135 (40.8)
LAD	113 (34.1)
CX	49 (14.8)
LM	34 (10.3)

BMI, body mass index; LV, left ventricle; ICD, implantable cardioverter defibrillator; CRT, cardiac resynchronisation therapy; CTO, chronic total occlusion; NSTEMI, non-ST-elevation myocardial infarction; UA, unstable angina; LM, left main stem.

**Table 2:** Outcomes in patients undergoing rotational atherectomy.

Outcome (*n* = 331)	% (*n*)
Emergency TPW insertion	0.3 (1)
Asystole	0.3 (1)
VF arrest	1.5 (5)
CPR	2.7 (9)
Death	2.4 (8)
Death related to RA-induced bradycardia	0.3 (1)
Tamponade	0.6 (2)
Stalled RA burr	0.6 (2)
Emergency CABG	1.2 (4)
Perforation	1.8 (6)
Coronary artery dissection	2.7 (9)
Aortic dissection	0.6 (2)
Vascular access complication	5.1 (17)
Contrast-induced acute kidney injury	2.1 (7)
Atropine use	6.0 (20)
Adrenaline use	0.9 (3)
Theophylline use	0
Isoprenaline use	0

Asystole was documented in one (0.3%) patient. Intravenous atropine was administered during procedure in 20 (6%) patients. Three (0.9%) patients received adrenaline. Only one (0.3%) patient received an emergency TPW. The presence of a permanent pacemaker or defibrillator (*n* = 22) may have masked symptomatic bradycardia induced by RA. Therefore, we calculated the need for emergency TPW insertion also separately for all patients without the back up of a permanent device (*n* = 309). In the group without a permanent pacing system, the need for emergency TPW was 0.32% (1/309). Therefore, the result did not differ significantly when compared with all-comers, including patients with permanent pacing systems (rate of emergency TPW need 0.30%; 1/331).

Nine (2.7%) patients required cardiopulmonary resuscitation (CPR), 5 (1.5%) had ventricular fibrillation (VF) arrest, while eight (2.4%) patients died following RA. However, the death of only one (0.3%) patient (the same who received the emergency TPW) is likely to be directly related to RA-induced bradycardia. The patient who died underwent RA due to a severely calcified LAD, in the concomitant presence of a chronic occlusion of the RCA.

Other complications, such as stalled burr, coronary perforation, emergency CABG, or aortic dissection were cumulatively relatively rare (<3% in total). Vascular access complications were reported in 17 (5.1%) of the cases. Due to the low number of the primary outcome (bail-out TPW insertion; *n* = 1), no further statistical tests to identify predictors of TPW insertion were performed.

## Discussion

Rotational atherectomy is an important technique for calcium modification in complex PCI. Its use varies from <1% up to 10% of all PCIs in selected centres (5). In Japan (6) or the United Kingdom (UK), it is used in about 3% of all PCI procedures, whereas in Germany, in only approximately 0.8% of all PCI cases (9). In the UK, the number of RA procedures has been continuously declining since 2018, which is likely related to the introduction and continuous increase of intravascular lithotripsy (IVL) use over the last few years (10).

Our study shows that RA is performed in a high-risk population with almost half of all patients (47.7%) being diabetic and/or having a history of smoking or obesity—with a significant proportion of patients having chronic kidney disease (CKD) (21%), peripheral artery disease (PAD) (20%), and advanced coronary artery disease (92% of cases had multivessel disease). More than one-third of all patients presented with an acute coronary syndrome (ACS) or ST-elevation myocardial infarction (STEMI). These characteristics are very similar to a ROTATE multicentre registry published by Kawamoto et al. (11). Female patients were previously shown to be older, have more frequent femoral access, and experience more net adverse clinical events compared with male patients undergoing RA PCI (12).

In our centre, the rate of radial access for most operators ranges between 85% and 90%. There was a high proportion of femoral access (48%) in the RA group, which was likely caused by two factors. First, before 2013 all procedures were performed by femoral access, reflecting the historical practice in Austria at that time. This theory would be supported by a sub-analysis of our centre's RA PCI procedures since the year 2020, when the rate of femoral access use decreased to 27% and the vast majority of procedures were performed using the trans-radial route. A multicentre study from UK enrolling 518 patients treated with RA between 2005 and 2013 confirmed a similar notion when at the time only 30.7% of all RA procedures were performed using the trans-radial approach (13).

Second, as mentioned previously, the population that undergoes RA is per definition a high-risk population with substantial prevalence of PAD and CKD, which likely affects the peripheral access options. The nature of heavily diseased coronary arteries, better support, and the need to accommodate bigger RA burrs frequently necessitates larger guides (e.g., 7 or 8 Fr) and hence femoral access, compared with non-RA PCI.

The main finding of our study is that despite the occasionally documented transient bradycardias, which necessitated the use of pharmacological treatment (atropine was used in 6% of patients), the need for emergency TPW insertion was exceedingly rare. Only one (0.3%) patient required emergency TPW. Unfortunately, this patient suffered cardiac arrest and died, and RA-induced bradycardia may have played an important role in this death. In-hospital mortality occurred in 2.7% (*n* = 8) of the patients. The vast majority of the deaths (except one) were not related to bradycardia induced by the RA procedure. Likewise, all other intraprocedural complications were very rare as previously shown by our data. Periprocedural and in-hospital mortality in our study compare unfavourably with mortality seen in elective uncomplicated PCI cases, which is usually below 1% [British Cardiovascular Intervention Society (BCIS) UK 0.2%]; however, it is in line with deaths reported for all PCI (2.3%) or primary PCI (6.2%) [PCI mortality data in brackets taken from BCIS audit 2021/2022 (10)]. Eftychiou et al. reported that in a population of patients treated with RA, the presence of PAD, diabetes mellitus (DM), ACS, and SYNTAX score ≥23 were all independent predictors of major adverse events (13). Pharmacological bail-out strategies were occasionally needed in our population (atropine in 6% and adrenaline in 0.3%) to treat significant bradycardias. Theophylline and aminophylline were not used in our institution, although they are occasionally used in other institutions to treat or prevent RA-related bradycardia. Acar et al. demonstrated in a small open label study (*n* = 60) that a reduction of bradycardia and heart block with a prophylactic aminophylline added to the heparin and nitroglycerin saline flush solution (rotaphilline) when compared with the heparin and nitroglycerine saline only flush solution (4).

Our finding of the exceedingly rare requirement for bail-out emergency temporary pacing implantation (0.3%), in an unselected real-life population undergoing RA, supports no routine insertion of a TPW prior to RA. Our findings are also reflected by the clinical experience of other operators over the last decade and increasingly prevalent expert consensus (currently not based on any data) that routine temporary wire insertion prior to RA is not necessary (5, 9).

The rare need for TPW appears in contrast to the findings of Mitar et al. (3), where 50% of patients had TPW inserted as a primary prevention measure. The authors reported that 31% of the patients developed higher-degree AV blocks or experienced TPW activation during the RA procedure. Whereas a transient AV block or bradycardia is fairly common during RA, in our experience, usually non-invasive vagolytic manoeuvres are sufficient to treat the vast majority, if not all, of these situations. In our single-centre study, there was only one case of emergency TPW insertion, which was related to bradycardia induced by RA. This particular patient died during the procedure. However, retrospectively it was difficult to establish the degree to which the bradycardia was related to the outcome. The small benefit of TPW has to be weighed against its potential risks that are potentially lethal such as RV wall perforation, cardiac tamponade, and major vascular access complications. In other words, the theoretical number-needed-to-treat (NNT) appears to be very high for the prophylactic TPW. A recent review by Tjong et al. reported that transvenous pacing for a variety of clinical scenarios was associated with a mean complication rate of 22.9%, out of which 5.7% were considered clinically significant (14). Another consideration is the additional procedural time and the material cost associated with routine prophylactic TPW insertion.

**Figure 1:**
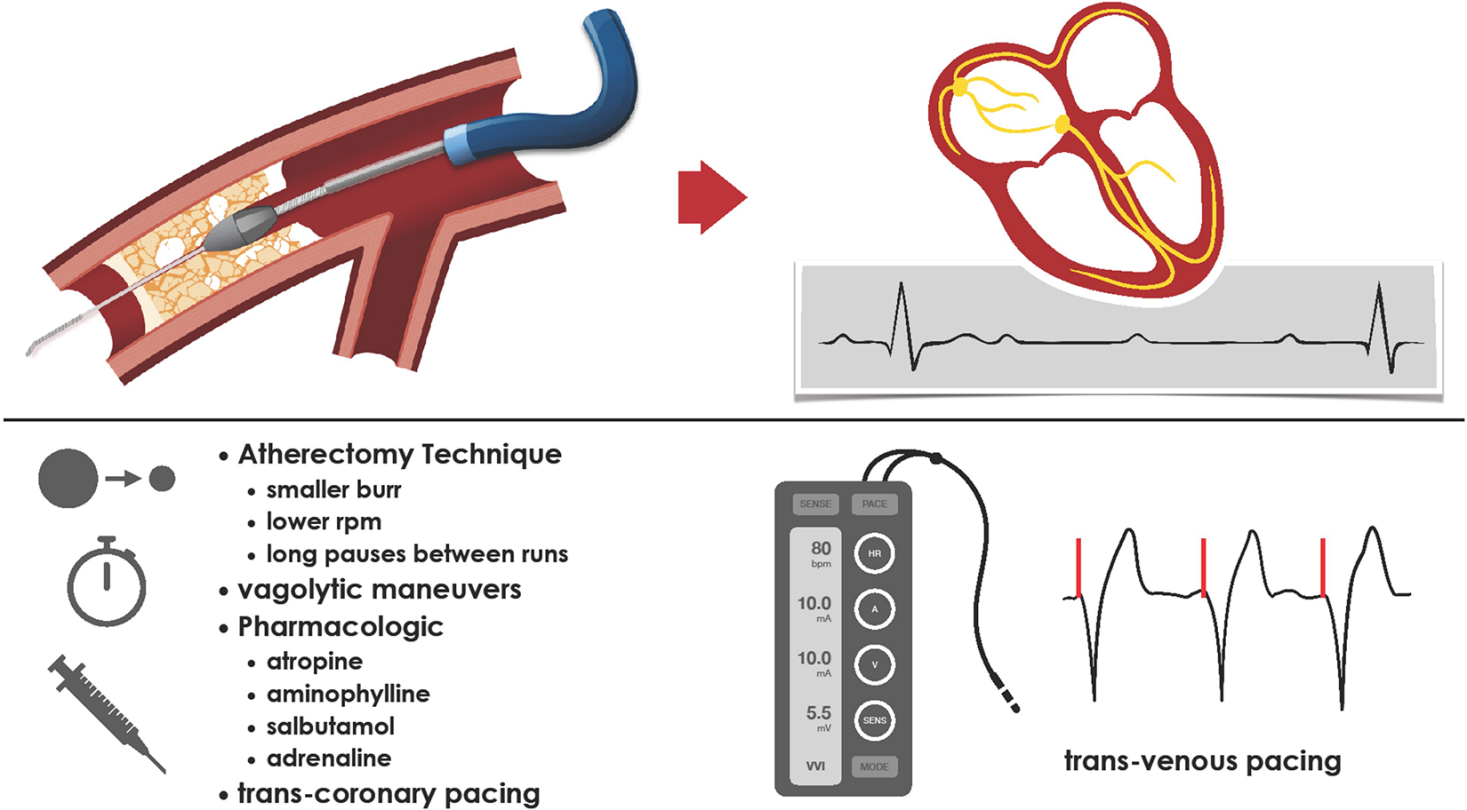
Rotational atherectomy-induced microembolisation can induce reflective bradycardia and transient heart block. There are several procedural aspects, which can limit the occurrence or impact of symptomatic bradycardia.

There are several techniques that can minimise the risk of significant bradycardias, or provide alternative, less invasive strategies compared with TPW insertion (Figure 1). These include the use of smaller burrs at the beginning, use of lower rotational speed, sufficient pauses between individual runs, vagolytic manoeuvres (coughing), pharmacology (atropine, adrenaline, aminophylline), and safety setups including routine external pacing pads and washed femoral access for emergency bail-out TPW insertion if needed (4, 15). An important role to minimise major adverse events associated with RA is played by the experience of the operator. Kinnaird et al. demonstrated that in-hospital adverse outcomes occurred less frequently as RA PCI operator volume increased (16).

Another interesting alternative to TPW pacing is the trans-coronary pacing (TCP) over the Rota wire, which was first described by Meier et al. in 1985 (17, 18). Recently, Iqbal et al. reported results of prophylactic TCP in 132 patients from the ROTA-PACE study. Successful TCP was reported in 121 (91.7%) patients and no complications were registered (19). The ROTA-PACE technique was not successful in 11 patients, of which in four the Rota wire could not be placed distally and four patients had large infarcts in the territory of the Rota wire. A further development of the trans-coronary pacing is the use of the Electroducer Sleeve developed recently by Benjamin Faurie. This is a special electro-conductive sheath that is supposed to standardise the direct wire pacing technique (both for TAVI or PCI) with more reproducibility and acceptable thresholds (20).

Future prospective randomized studies are warranted to investigate the safety and efficacy of systematic use of prophylactic pharmacology together with trans-coronary pacing vs. bail-out transvenous pacing vs. routine prophylactic pacing in patients undergoing RA PCI.

## Limitations

There are several limitations to this study. The main limitation is the retrospective single-centre nature of this study and the fairly long observational period. There is likely a wide variety of experiences of different operators with the RA procedure. This may have played an important role, especially in the first few years of the observational period. However, it is extremely reassuring that the bail-out need for routine transvenous pacing was exceedingly rare.

## Conclusion

We present that bail-out TPW is rarely needed during RA. A standby temporary pacing strategy is reasonable and may avoid unnecessary procedural complications when compared with the routine prophylactic transvenous pacing strategy. Multiple non-invasive strategies and optimisation of the RA technique may minimise the risk of clinically significant bradycardias during RA. An individualised approach with TPW insertion or the use of trans-coronary pacing using the Rota wire in very high-risk patients may offer reasonable alternatives to routine TPW insertion.

## Data Availability

The datasets presented in this study can be found in online repositories. The names of the repository/repositories and accession number(s), that is, a preprint of an older version of the data can be found at https://www.medrxiv.org/content/10.1101/2023.10.12.23296980v1.
